# A Different Exosome Secretion Pattern Characterizes Patient-Derived Colorectal Cancer Multicellular Spheroids and Their Mouse Xenografts

**DOI:** 10.3390/biology11101427

**Published:** 2022-09-29

**Authors:** Michela Relucenti, Federica Francescangeli, Maria Laura De Angelis, Vito D’Andrea, Selenia Miglietta, Orlando Donfrancesco, Xiaobo Li, Rui Chen, Ann Zeuner, Giuseppe Familiari

**Affiliations:** 1 Section of Human Anatomy, Department of Anatomical, Histological, Forensic Medicine and Orthopedic Sciences, Sapienza University of Rome, 00161 Rome, Italy; 2 Department of Oncology and Molecular Medicine, National Institute of Health (Istituto Superiore di Sanità), 00161 Rome, Italy; 3 Department of Surgical Sciences, Sapienza University of Rome, 00161 Rome, Italy; 4 Beijing Key Laboratory of Environmental Toxicology, Capital Medical University, Beijing 100069, China

**Keywords:** colorectal cancer, exosomes, transmission electron microscopy, scanning electron microscopy, spheroid, xenograft

## Abstract

**Simple Summary:**

Exosomes have a role in tumorigenesis and metastatic dissemination, their material content and size being associated with poor prognosis of colorectal cancer (CRC). Our work aims to investigate their secretion patterns in CRC stem cells in patient-derived multicellular tumor spheroids (MTSs) and their mouse xenografts, to unveil possible differences in terms of exosome amount, size, and secretion site between in vitro and in vivo models. Our results show that MTSs’ exosome secretion pattern depends on their structural complexity: few-layer spheroids show a lesser exosome secretion, limited to the apical domain of cancer cells; secretion increases in multilayered spheroids and is visible from apical and basolateral cancer cells domains. In xenograft models, exosome secretion occurs from all cancer cell domains, and it is quantitatively greater than that observed in spheroids. The influence of the surrounding environment of non-tumor cells may account for the difference in exosome secretion patterns between spheroids and xenografts.

**Abstract:**

Up-to-date in vitro and in vivo preclinical models expressing the patient-specific cancer lineage responsible for CRC and its metastatic behavior and responsiveness to therapy are needed. Exosomes’ role in tumorigenesis and the metastatic process was demonstrated, and the material content and size of the exosomes are associated with a poor prognosis of CRC. Exosomes are generally imagined after their recovery from blood serum as isolated entities, and our work aims to investigate them “in situ” in their native environment by scanning and transmission electron microscopy to understand their secretion modalities. We studied CRC stem cells in patient-derived multicellular tumor spheroids (MTSs) and in their mouse xenograft to find possible differences in terms of exosome amount, size, and secretion site between in vitro and in vivo models. We observed that MTSs’ exosome secretion patterns depend on their structural complexity: few-layer MTSs show a lesser exosome secretion, limited to the apical domain of cancer cells, secretion increases in multilayered MTSs, and it develops from apical and basolateral cancer cells domains. In xenograft models, exosome secretion occurs from all cancer cell domains, and it is quantitatively greater than that observed in MTSs. This difference in exosome secretion pattern between MTSs and xenografts may be due to the influence of surrounding non-tumor cells.

## 1. Introduction

Epidemiological data on colorectal cancer (CRC) incidence and mortality (according to the World Health Organization GLOBOCAN database) show that CRC is the third most commonly diagnosed cancer in males and the second in females around the world, even if marked differences in rates exist among countries [1]. Stand-alone surgery is generally curative in 40% of patients with CRC stages 1 or 2, and 5-year survival rates reach 90% [2]. This approach is not sufficient for the management of advanced stages and metastatic CRC, which represent about 30% of cases at the time of diagnosis [3]. For this kind of patient, chemotherapy, radiotherapy, and their combination are used, but a marked heterogeneity in patients’ clinical responses exists [4,5], accounting for poor survival rates [6].

One strategy to reduce mortality in CRC is, on the one hand, to succeed in finding biomarkers for early detection and, on the other, the development of personalized therapies to treat patients in the more advanced stages of the disease. The oncology community is moving towards patient-tailored cancer therapy, taking into account the unique molecular profile of each patient’s cancer. This innovative approach is improving responses to therapy [7,8], and the development of up-to-date in vitro and in vivo preclinical models expressing the patient-specific cancer lineage and genetic diversity is needed to understand some fundamental aspects of patient-specific genetic alterations, not only in CRC arising but moreover in its metastatic behavior and responsiveness to therapy. In the last two decades, MTSs cultures have been developed from CRC and other tumors and are now considered reliable preclinical in vitro models of cancer [9]. CRC MTSs consist of 3D cultures of primary cells derived from surgical specimens, reproducing patient-specific genetic expression profiles and heterogeneity [10].

At the same time, the role of exosomes as specific biomarkers in CRC prediction and screening is emerging [11]. Exosomes are nano-sized vesicles (30–120 nm), in their single membrane, express a high and cancer-specific glycosylation profile [12] and are the carrier for various lipids, proteins, DNA fragments, and several RNA species as mRNAs, microRNAs (miRNAs), long noncoding RNAs (lncRNAs) as well as small interfering RNAs (siRNAs) [13,14,15,16,17]. The potential role of exosomes in tumorigenesis and the metastatic process was demonstrated [18,19,20]. Exosomes’ role in cancer progression and metastasis is that of carriers, which actively transfer bioactive molecules between cancer cells and different cell types in the nearby and distant microenvironments. The effect of such intercellular cross-talk explicates by changing multiple cellular and biological functions in recipient cells [21,22,23]. Moreover, the poor prognosis of cancer is associated with the material content and the size of the exosomes rather than the frequency of blood circulating exosomes. Exosomes are generally imaged after their recovery from blood serum by drop-casting as isolated entities, and we aim to look at them in their native environment. We focused on an exosome secretion pattern study “in situ,” observed by scanning and transmission electron microscopy patient-derived MTSs and their mouse xenograft, to find possible differences in terms of exosome amount, size, and secretion site between in vitro and in vivo models.

## 2. Materials and Methods

### 2.1. CSC MTSs Isolation and Culture

- Patient

A 63 aged year male underwent CRC surgery for cancer removal under the standards of the ethics committee on human experimentation of the National Institute of Health (Istituto Superiore di Sanità) authorization no.CE5ISS 09/282, as reported in [24].

- Cancer biopsies management (immediately after recovery)

Samples were washed 2–3 times in cold saline and transferred in Dulbecco’s modified Eagle’s medium (DMEM; Thermo Fisher Scientific, Carlsbad, CA, USA, https://www.thermofisher.com, accessed on 27 May 2022), mixed with 3% penicillin-streptomycin-amphotericin B solution (Lonza Group, Walkersville, MD, USA, http://www.lonza.com, accessed on 27 May 2022).

- Biopsies dissociation procedure

Samples were washed 3–4 times in phosphate-buffered saline (PBS), sectioned into small fragments (0.5 × 0.5 mm), and incubated in DMEM (Thermo Fisher Scientific) with 1.5 mg/mL collagenase type II (Thermo Fisher Scientific) and 20 g/mL DNAse (Roche Diagnostics, Indianapolis, IN, USA, https://usdiagnostics.roche.com, accessed on 27 May 2022) for 1 h at 37 °C, under shaking.

- Cell culture

Resuspensions of pellets containing cells, cell clusters, and tissue fragments were cultured in CSC medium supplemented with 10 mm nicotinamide, 1 µm y-27632 (both from Sigma-Aldrich, St. Louis, MO, USA, http://www.sigmaaldrich.com, accessed on 27 May 2022), 20 ng/mL human EGF and 10 ng/mL human basic fibroblast growth factor (both from Peprotech, London, UK, https://www.peprotech.com, accessed on 27 May 2022). For further detail, see [10,24].

### 2.2. Animal Procedures

Animal procedures were performed according to the Italian National animal experimentation guidelines (D.L.116/92) and upon approval of the experimental protocol by the Italian Ministry of Health’s Animal Experimentation Committee.

- Animals

Four- to 6-week-old female NOD.Cg-Prkdcscid Il2rgtm1Wjl/SzJ (NSG) mice (The Jackson Laboratory, Bar Harbor, ME, USA, https://www.jax.org, accessed on 27 May 2022). For CSC validation, 5 × 105 cells were injected subcutaneously in the flank of 3 replicate mice in 100 µL 1:1 PBS/Matrigel (BD, Franklin Lakes, NJ, USA, http://www.bd.com, accessed on 27 May 2022). In all the validated CSCs, xenografts were detectable within 3–5 weeks in at least 2/3 mice.

- Xenograft extraction and treatment

Palpable xenografts were extracted, and samples were then formalin-fixed and paraffin-embedded. A pathologist evaluated hematoxylin and eosin-stained sections to compare xenograft histology with that of the tumor of human origin.

### 2.3. Scanning Electron Microscopy (SEM) Protocol for MTSs

- Primary fixation (immediately upon recovery)

MTSs were fixed in a solution of 2.5% glutaraldehyde in Phosphate buffer solution 0.1 M, pH 7.4 at 4 °C for 48 h.

- Washing: samples were rinsed overnight in Phosphate buffer solution 0.1 M, pH 7.4 at 4 °C.
- Post-fixation

A solution of osmium tetroxide (OsO_4_) at 1.33% in H_2_O (Agar Scientific, Stansted, UK) was used to submerge tissue fragments for 2 h.

- Washing: phosphate buffer solution 0.1 M, pH 7.4 for 20 min (10 + 10 min) was used to remove (OsO_4_) residuals [25,26,27].
- Dehydration procedure

Ascending alcohol series (30, 70, 95, and 100% *v*/*v*) solutions were used.

- Critical point drying procedure (Emitech K850, Emitech, Corato, Italy).
- Samples were mounted on aluminum stubs using carbon tape.
- Sputter coating procedure.

With platinum (Emitech K 550 sputter coater, Emitech, Corato, Italy operating conditions: 15 mA, for 3 min).

- Observation

Hitachi SU 4000 Field emission scanning electron microscope under high vacuum at 20 kV. Digital image acquisition system: DISS5 Digital Image Scanning System (Point Electronic, Halle (Saale), Germany).

### 2.4. Transmission Electron Microscopy (TEM) Protocol for MTSs and Xenograft

- Primary fixation (immediately upon recovery)

MTSs and xenograft biopsies were fixed in a solution of 2.5% glutaraldehyde in phosphate buffer 0.1 M, pH 7.4 at 4 C for 48 h.

- Washing: samples were rinsed overnight in Phosphate buffer solution 0.1 M, pH 7.4 at 4 °C.
- Post-fixation

Samples were then post-fixed in a solution of OsO_4_ 1.33% in H_2_O (Agar Scientific, Stansted, UK) for 2 h.

- Washing: phosphate buffer solution 0.1 M, pH 7.4 for 20 min (10 min + 10 min) was used to remove (OsO_4_) residuals.
- Dehydration procedure

Ascending alcohol series (30, 70, 95, and 100% *v*/*v*) solutions were used.

- Substitution procedure

Propylene oxide was used (BDH Italia, Milan, Italy), 2 steps of 20 min each.

- Embedding procedure

In a mixture of 50:50 propylene oxide and epoxy resin Agar 100 (SIC, Rome, Italy) overnight at 25 °C (under a chemical fume hood). Finally, samples were embedded in fresh epoxy resin Agar 100 (Agar scientific, Agar Scientific Ltd., Stansted, Essex, UK) and put on a stove at 60 °C for 48 h [28,29,30].

Sectioning procedure:

- Semithin sections (1 m thick) were collected on glass slides, stained blue by methylene blue, to perform light microscopy observations by a Zeiss Axioskop-40 (Carl Zeiss, Oberkochen, Germany) equipped with Axiovision image acquisition software.
- Ultrathin sections for TEM observations were cut using an ultramicrotome (Leica EM UC6, Vienna, Austria). Ultrathin sections were collected on 100-mesh copper grids (Assing, Rome, Italy). Staining was performed using Uranyless© solution and lead citrate 3% solution (Electron Microscopy Science, 1560 Industry Road, Hatfield, PA, USA).

Imaging procedure:

- Observation under a transmission electron microscope (Carl Zeiss EM10, Thornwood, NY, USA) set with an accelerating voltage of 60 kV.
- Digital image acquisition system: CCD digital camera (AMT CCD, Deben UK Ltd., Suffolk, UK).

### 2.5. Exosome and Multivesicular Bodies (MVBs) Size Measurement and Statistical Analysis

Exosomes and MVBs diameters (N = 200 for each group: spheroid apical, spheroid basolateral, xenograft apical, xenograft basolateral, MVB Apical, MVB basolateral) were measured on transmission electron microscopy digital images using open source Fiji software [31] and Hitachi 3D Map (Digital Surf, Besancon, France) [32]. Data were statistically analyzed, and summary statistics, *t*-tests, and ANOVAs with Bonferroni correction were performed, and data were plotted in histograms. All procedures were performed using Med Calc Statistical software (MedCalc Software 20.009 version Ltd., Acacialaan 22, 8400 Ostend, Belgium).

## 3. Results

Our morphological investigation started with the analysis of the MTSs’ three-dimensional morphology by scanning electron microscopy; we then proceeded to their ultrastructural characterization; by transmission electron microscopy, highlighting aspects related to the secretion of exosomes. Finally, an ultrastructural analysis of the xenograft was performed to compare the different experimental models and highlight similarities and differences in exosome secretion. Data on the size of exosomes and MVBs were then finally statistically analyzed.

### 3.1. SEM Analysis of MTSs Morphology and Exosomes Secretion

**Figure 1:**
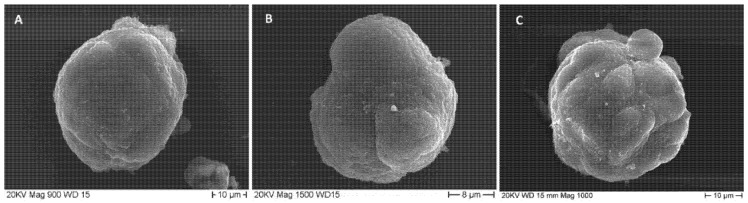
In vitro cultured MTSs were observed by scanning electron microscopy. (**A**) sample with a smooth outer surface is shown, magnification 900×, bar 10 µm. (**B**) Sample with superficial and shallow furrows is represented, magnification 1500×, bar 8 µm. (**C**) Spheroid with outer surface marked by deep and numerous grooves, cell borders are evident, and one cell stands out from the spheroid mass, magnification 1000×, bar 10 µm.

Observation of the outer morphology of MTSs by scanning electron microscopy showed that their outer surface had a variable appearance. Some MTSs appeared as compact entities with smooth surfaces or sparse, shallow furrows (Figure 1A). No exosome secretion was observed from the cells forming the outer surface. Other MTSs showed some shallow surface grooves (Figure 1B), corresponding to the boundaries of the underlying cells; even in this case, no exosome secretion by the outermost cells was observed. Still, other MTSs exhibited on their outer surface deep and numerous grooves (Figure 1C), with one or more cells protruding from the surface of the spheroid itself. The cells of the outermost layer exhibit blebs and microvilli, but no exosome secretion was observed.

### 3.2. LM (Light Microscopy) and TEM Analysis of MTSs Cells Morphology and Exosomes Secretion

**Figure 2:**
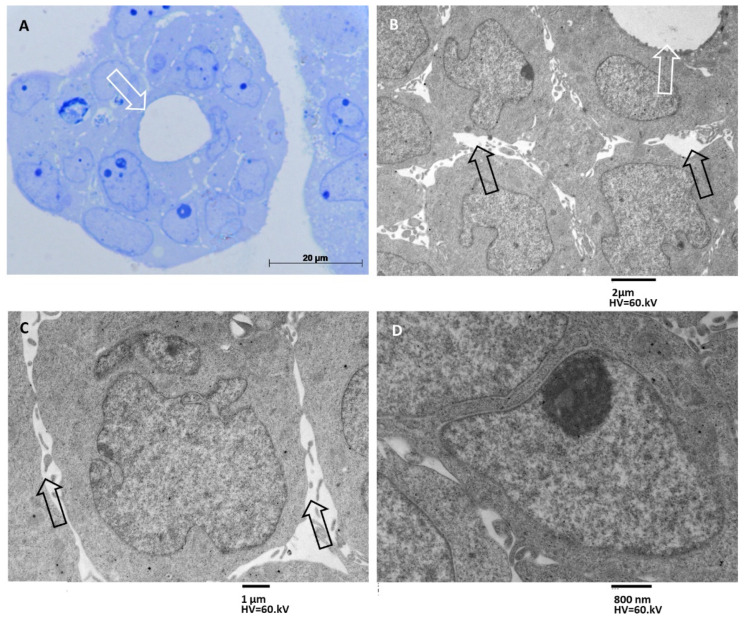
MTSs of Group A. (**A**) Light microscopy image of a bi-layered spheroid containing a pseudocyst-like structure. Magnification 400×, bar 20 µm. (**B**) TEM image. Cells lining the lumen (white arrow) of the pseudocyst are tightly adherent to each other, and no exosome secretion in the lumen of the pseudocyst was visible. Magnification 1250×, bar 2 µm. (**C**) TEM image. Cells in the outermost layers appear loosely adherent to each other and separated by large intercellular spaces (black arrows) in which cells’ membrane finger-like extroversions extend. Magnification 1600×, bar 1 µm. (**D**) Finger-like membrane extroversions were sometimes short and, at other times, were longer and convoluted, intertwining with those of adjacent cells. No exosome secretion in the intercellular spaces was visible. Magnification 1800×, bar 800 nm.

Observing the internal morphology of the MTSs, at first by light microscopy on semi-thin sections, then by transmission electron microscopy on ultrathin sections, we identified three different types of MTSs, hereafter defined as A, B, and C. MTSs of group A were characterized as bi-layered or three-layered structures, containing pseudocyst-like structures (resembling a colonic gland; Figure 2A). Cells lining the pseudocyst lumen tightly adhere to each other and project microvilli on their apical surface. In the pseudocyst lumen, no exosome secretion was visible. The cells of the outermost layers appeared loosely adhered to each other and were separated by large intercellular spaces (Figure 2B,C) in which cells’ membrane extroversions extended (Figure 2C,D). These threadlike extroversions were sometimes short, other times were longer and convoluted, intertwining with those of adjacent cells. In the intercellular spaces, no exosome secretion was visible.

**Figure 3:**
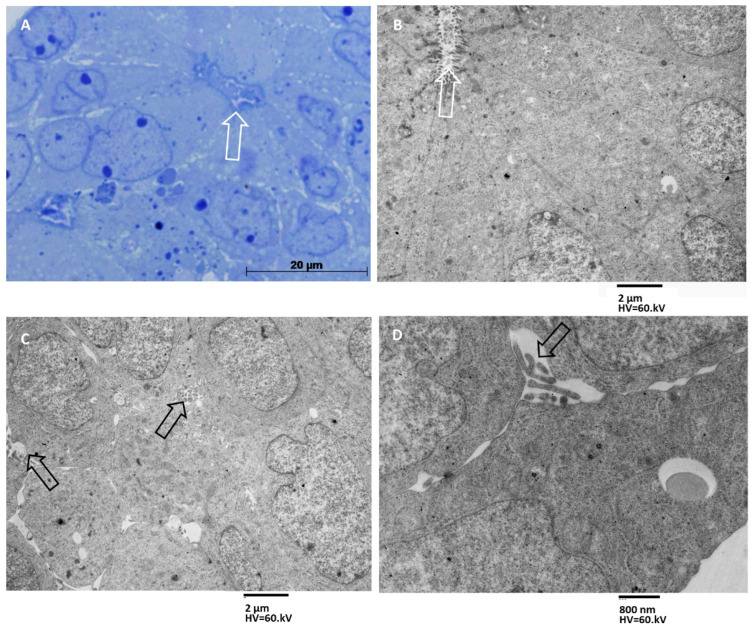
MTSs of group B. (**A**) Light microscopy image of a three-layered spheroid containing a pseudocyst-like structure (lumen pointed at by an arrow). Magnification 400×, bar 20 µm. (**B**) TEM image shows that cells lining the lumen of the pseudocyst are tightly adhered to each other, possess microvilli on the apical surface, and secrete exosomes in the lumen (white arrow). Magnification 1250×, bar 2 µm. (**C**) Cells of the outermost layers are adherent to each with just narrow intercellular spaces (black arrows). Magnification 1250×, bar 2 µm. (**D**) Finger-like cells’ membrane eversions into the narrow intercellular spaces are shown, and no exosome secretion is present. Magnification 4000×, bar 800 nm.

Group B MTSs were structured in three to five cell layers, and the innermost cells were arranged to form a pseudocyst-like structure (Figure 3A). Cells lining the lumen of the pseudocyst tightly adhered to each other and presented microvilli on the apical surface and secrete (Figure 3B). Cells in the outermost layers appeared more adherent to each other than in the same cells of group A MTSs, but intercellular spaces are still present (Figure 3C). These cells also project finger-like membrane eversions into the intercellular spaces, but we did not observe the secretion of exosomes into these spaces (Figure 3C) or towards the external surface of the spheroid.

**Figure 4:**
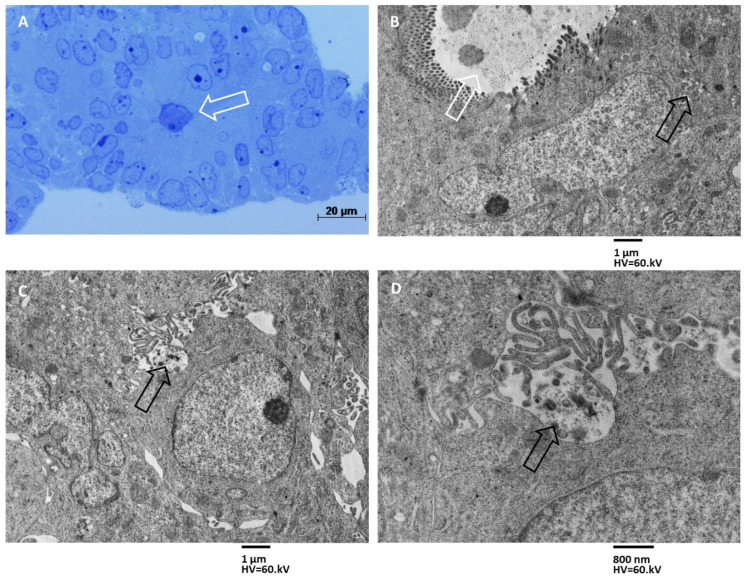
MTSs of group C. (**A**) Light microscopy image of a multilayered spheroid containing a pseudocyst-like structure, the lumen is indicated with a white arrow. Magnification 400×, bar 20 µm. (**B**) Cells lining the pseudocyst lumen had microvilli on the apical surface and secreted a large number of exosomes (white arrow). Cells are tightly adherent in their apical region, while in the basolateral domain, some intercellular spaces (black arrow) are visible. Magnification 1600×, bar 1 µm. (**C**) Cells in the outermost layers are separated by rare and narrow intercellular spaces, in which finger-like eversions of cell membranes project (black arrow). Magnification 1600×, bar 1 µm. (**D**) Secretion of exosomes was observed from the middle layers of cells’ membrane, which spill exosomes into the intercellular spaces (black arrow). No exosome secretion was observed from the outermost cell layers towards the external surface of the spheroid. Magnification 4000×, bar 800 nm.

Group C MTSs have been identified as multilayered structures whose innermost cells arrange to form a pseudocyst-like structure (Figure 4A,B). The cells bordering the lumen of the pseudocyst were tightly adhered to each other, possessed microvilli on the apical surface, and secreted a large number of exosomes (Figure 4B) and entire MVBs.

The cells of the outermost layers were also in contact with each other (Figure 4B), few intercellular spaces were present, and their lumen was occupied by digitiform eversions of cell membranes. In this group of MTSs, the same cells with microvilli that excrete exosomes into the lumen of the pseudocyst can also secrete exosomes from the membrane of the lateral domain, and the secretion of exosomes was observed from the middle layers cells’ membrane, which spills exosomes into the intercellular spaces (Figure 4C). No exosome secretion was observed by cells’ membrane of outermost layers towards the external surface of the spheroid, i.e., directly in the culture medium.

### 3.3. LM and TEM Analysis of Xenograft Morphology and Exosomes Secretion

**Figure 5:**
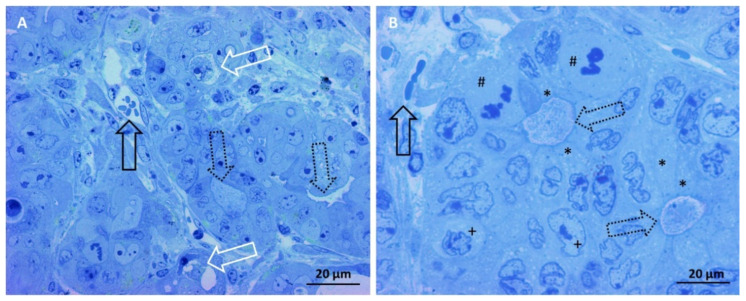
Xenograft tissue organization is illustrated by LM, magnification 400×. (**A**) The xenograft tissue consists of pseudocyst structures (black dotted arrows) arranged into a scarce stroma. The tissue is vascularized (black arrow), but hypoxic and necrotic areas are visible (white arrows). (**B**) Cells with columnar shape, similar to enterocytes (*), line pseudocyst lumen. Round cells with large oval and/or indented nuclei (+) do not reach the gland lumen. Mitotic figures (#) are observed, and blood vessels are shown (black arrow).

The tumor resulting from cancer-derived MTSs xenograft in immunodeficient mice (for simplicity, we will call this the xenograft) is a tissue, a more complex entity with different characteristics than MTSs, which are a 3D cultured cell mass. Simply consider the presence of necrotic and hypoxic areas, blood vessels, nerves, and fibroblasts (Figure 5A); altogether, those factors create a different extracellular environment that is lacking in the MTSs culture system. Xenograft contains cells arranged often in pseudocysts resembling crypts and glands of the colonic tract (Figure 5A,B) with scarce stroma in between. No goblet or enteroendocrine cells were visible as in MTSs from whom the xenograft originated or in the patient’s cancer. Those pseudocysts contained cells with a columnar shape similar to enterocytes, as well as more oval cells with large oval and indented nuclei, which did not open into the gland lumen and mitotic figures. Cell nuclei were dysmorphic, with large nucleoli and heterochromatin aggregates along the inner aspect of the nuclear membrane.

**Figure 6:**
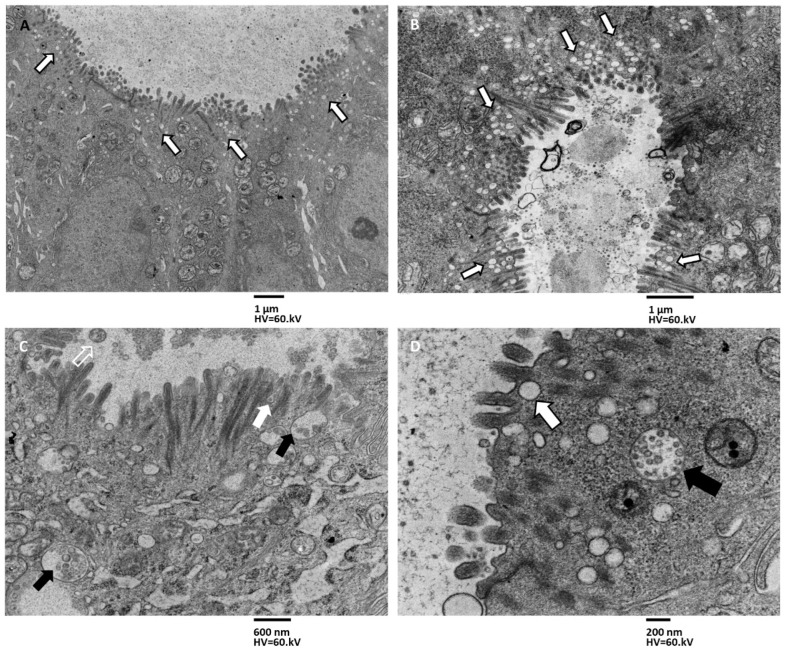
TEM images of exosomes secretion pattern in xenografts, apical surface secretion. (**A**) Numerous MVBs crowd the apical portion of pseudocyst lining epithelial cells, magnification 1600×, bar 1 µm. (**B**) MVBs accumulate just beneath the apical membrane, aligning in rows (arrows); the cyst lumen is filled with exosomes, with a magnification of 1600×, bar 1 µm. (**C**) MVBs (black arrows) travel across the cytoplasm, reaching the cell membrane and creeping into the narrow spaces among the microvilli base. Then they fuse with the plasmatic membrane (with arrow) and free their content into the cyst lumen. Sometimes entire MVBs are secreted (with an empty arrow), magnification 6300×, bar 600 nm. (**D**) At higher magnifications, MVBs filled with exosomes are visible (black arrow), and others approaching the plasmatic membrane are present (white arrow), magnification 12,500×, bar 200 nm.

To study the secretion patterns of exosomes in xenografts, images obtained by transmission electron microscopy observation of the samples were analyzed. The TEM images shown in Figure 6 demonstrate the presence of secretion activity in the apical domain of cells with microvilli surrounding the lumen of the pseudocyst. This secretion activity is intense and develops homogeneously along the apical surface. The intensity of secretion is caused by the presence of numerous MVBs (Figure 6A–C), aligned in rows perpendicular to the apical surface of the cell (Figure 6A–C). The MVBs release their exosome content at the base of the microvilli (Figure 6C,D), and MVBs are often entirely secreted.

**Figure 7:**
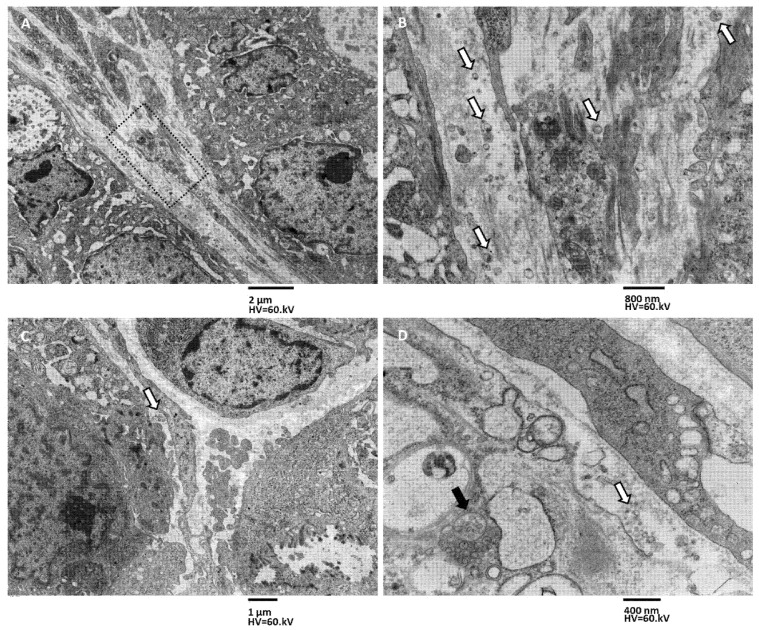
TEM images of exosome secretion pattern in xenografts, basolateral surface secretion. (**A**) An area of xenograft tissue stroma, between pseudocyst lining epithelial cells (on the right and top corner) and deeper tissue cells (on the left bottom corner), is illustrated (the dotted rectangle represents the area of panel (**B**)), magnification 1250×, bar 2 µm. (**B**) Magnification of dotted rectangle area in panel (**A**). Free exosomes (arrows) are visible in the scarce and loose stroma; they are seen both near the basolateral surface of epithelial-like cells (on the right) and near the deeper cells membrane (on the left), magnification 4000×, bar 800 nm. (**C**) Interstitial secretion of deeper cells is illustrated, free exosomes (arrow) are visible, magnification 1600×, bar 1 µm. (**D**) Deeper cells contain MVBs (black arrow) that fuse with the membrane and free exosome (with arrow) in the interstitium magnification 8000, bar 400 nm.

Phenomena of exosome secretion were also observed from the basolateral domain of xenograft cells facing the tissue interstitium (Figure 7). In particular, cells with microvilli were observed secreting exosomes into the lumen of pseudocyst from their apical domain but also towards the tissue interstitium from the basolateral domain (Figure 7A,B). This secretory activity is less intense than that along the apical surface. This lower intensity is caused by the fact that there are far fewer MVBs pouring their contents into the intercellular space, and these MVBs are scattered and not organized in parallel rows as they were in the apical domain. Secretory activity of exosomes was also observed from deeper cells that do not face into the lumen of the pseudocyst and that lack polarization in the apical, lateral, and basal domains (Figure 7C,D). No entire MVB secretion was observed from the basolateral cells’ domain.

**Figure 8:**
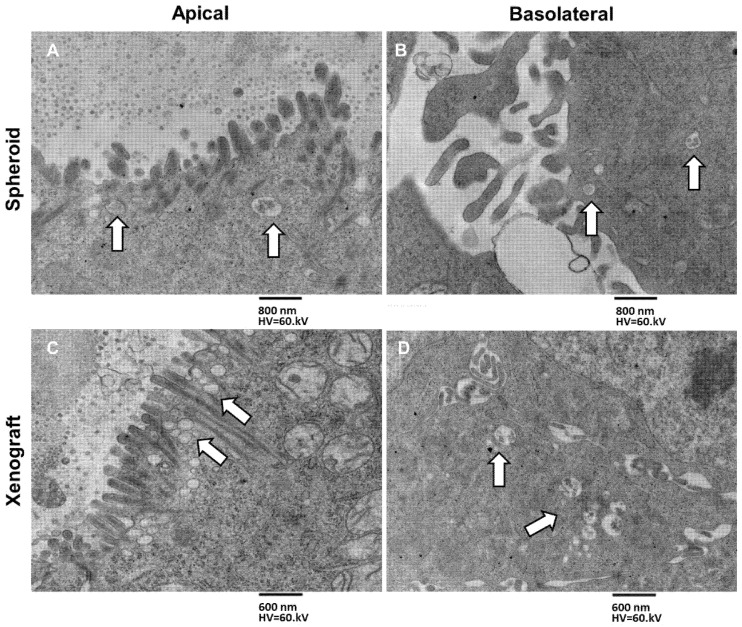
TEM images of exosome secretion pattern in MTSs and xenografts, apical vs. basolateral surface secretion. (**A**) MVBs secretion from spheroid apical surface, arrows indicate MVBs accumulating just beneath the apical membrane, aligning in rows, 6300×, bar 800 nm. (**B**) MVBs secretion from the spheroid’s basolateral surface, arrows indicate single MVBs approaching the basolateral surface, 6300×, bar 800 nm. (**C**) MVBs secretion from xenograft apical surface, arrows indicate massive MVBs accumulation just beneath the apical membrane. MVBs arrange in columns perpendicular to the apical membrane, magnification 6300×, bar 600 nm. (**D**) MVBs secretion from xenograft basolateral surface, groups of MBVs (arrows) accumulating just beneath the basolateral membrane, magnification 6300×, bar 600 nm.

The different patterns of secretion in MTSs and xenografts at both the apical and basolateral membranes, secretion in MTSs and xenograft is shown in Figure 8. In MTSs samples, few and sparse MVBs are aligned in one horizontal row just beneath the apical plasmatic membrane. In xenografts, MVBs are aligned in several vertical columns perpendicular to the apical plasmatic membrane (Figure 8A,B; see also Figure 6). Few MVBs approach the basolateral membrane in MTSs, while groups of generally four or five MVBs are visible near the basolateral membrane in the xenografts (Figure 8C,D).

**Figure 9:**
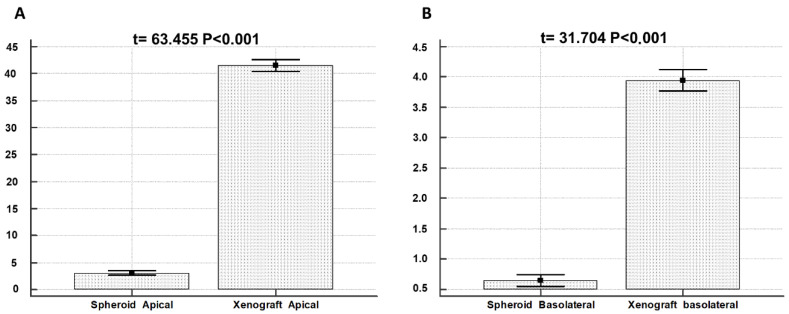
Comparison of MVBs secretion between spheroid and xenograft. (**A**) *t*-test results of spheroid vs. xenograft apical membrane secretion. (**B**) *t*-test results of spheroid vs. xenograft basolateral membrane secretion.

To provide a quantification of different secretion amounts in MTSs and xenografts, we counted the number of MVBs in epithelial cells lining pseudocystic structures (200 cells from Type C MTSs and 200 cells from xenografts). Summary statistics and *t*-test results are illustrated in Table 1 and Figure 9.

MBV secretion from the apical domain of spheroid cells is five times higher than basolateral secretion. MBV secretion from the apical domain of xenograft cells is 10 times higher than basolateral secretion. MBV secretion from the apical domain of xenograft cells is massive compared to secretion from the apical domain of spheroid cells, in a ratio of 13:1. MBVs secretion from the apical domain of xenograft cells is higher than secretion from the apical domain of spheroid cells, in a ratio of 6:1.

### 3.4. Analysis of Exosomes and MVBs Morphology and Size by Transmission Electron Microscopy

Following the observation of MTSs’ and xenografts’ different secretory modalities, we focused our analysis on the morphology and size of exosomes and MVBs.

**Figure 10:**
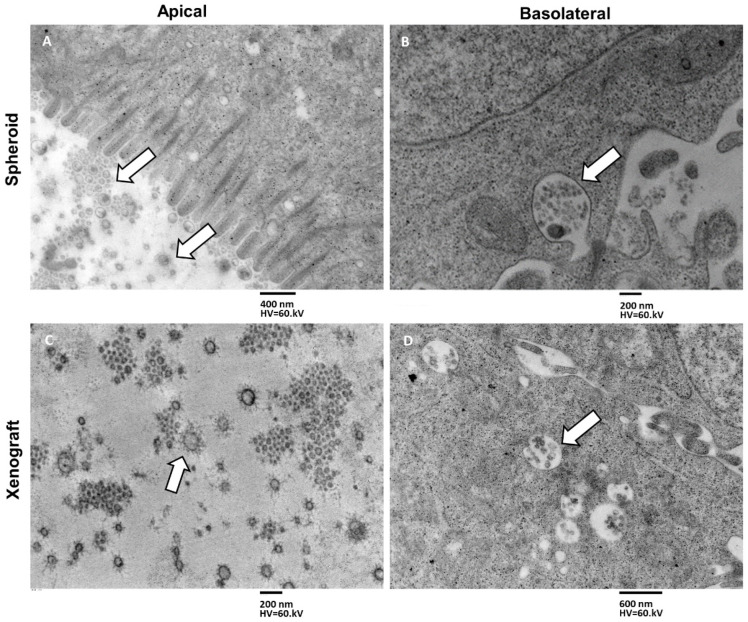
(**A**) Type C spheroid, apical surface secretion, exosomes (arrows) appear surrounded by a rich filamentous network, magnification 8000, bar 400 nm. (**B**) Type C spheroid, basolateral surface secretion, an MVB (arrow) is fusing with the basolateral membrane, and exosomes do not show the same well-developed filaments’ network of the apical-secreted ones, magnification 10,000, bar 200 nm. (**C**) Xenograft, apical surface secretion, exosomes (arrow) appear surrounded by a radial arrangement, well-developed filament’s network, magnification 125,000, bar 200 nm. (**D**) Xenograft, basolateral surface secretion, and several MVBs (arrow) are approaching the membrane. The exosomes contained appear not to have the same radially arranged rich filaments’ network observed in the apical-secreted ones, magnification 6300, bar 600 nm.

Looking at the appearance of exosomes secreted from the apical domain (in both MTSs and xenograft), we noted that they presented long filaments projecting radially from the outer aspect of the membrane. These molecules partly intertwine at their initial part, proximal to the membrane, and form a “crown” around the outer surface of the exosome. These filaments consist of glycoproteins and glycolipids of exosome membrane, highlighted by the use of tannic acid in the sample preparation process for electron microscopy (Figure 10A,C). Exosomes secreted from the basolateral domain (Figure 10B,D) also showed glycoproteins and glycolipids on their membrane, although in smaller amounts than in exosomes secreted from the apical domain (Figure 10C,D).

**Figure 11:**
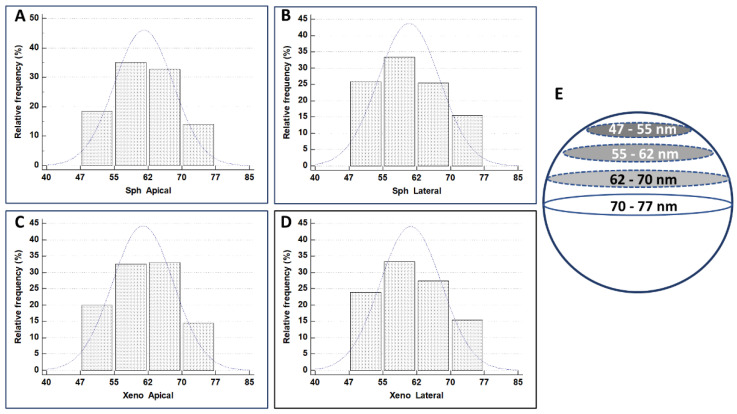
Histograms illustrate the distribution of diameter values in apical-secreted and basolateral-secreted exosomes in MTSs and xenografts. (**A**) Exosome diameter in spheroid apical secretion. (**B**) Exosome diameter in spheroid basolateral secretion. (**C**) Exosome diameter in xenograft apical secretion. (**D**) Exosome diameter in xenograft basolateral secretion. (**E**) The maximum diameter is the less frequent, and the others occur at least two times (over and above the equator).

Exosomes secreted in basal and inflammatory conditions from human epithelial cells had a size range of 30–90 nm [33,34]. We measured the size of exosomes secreted from the different domains in the same sample (spheroid apical vs. spheroid basolateral and xenotransplant apical vs. xenotransplant basolateral), and data were statistically analyzed (Table 2, Figure 11).

As can be seen from Figure 10C, the exosomes observed in a tissue sample included in resin and cross-sectioned appear not to have all the same size, as it is when vesicles extracted by centrifugation from culture medium or serum are observed at TEM by drop-casting. In the latter, the real diameter of the vesicles corresponds to the average value of the observed diameters. In our case, the correct interpretation of the real size of the vesicles must be made, taking into account that, since these are spherical dissected structures, the real diameter of the vesicle will correspond not to the average value of the observed diameters but will correspond to their maximum value (Figure 11). The distribution values show that values in the first three columns are about two times higher concerning the last column (70–77 nm), being the value of maximum diameter (sphere equator in Figure 11E) the lesser frequent for the values in the others classes, that recur at least two times.

**Figure 12:**
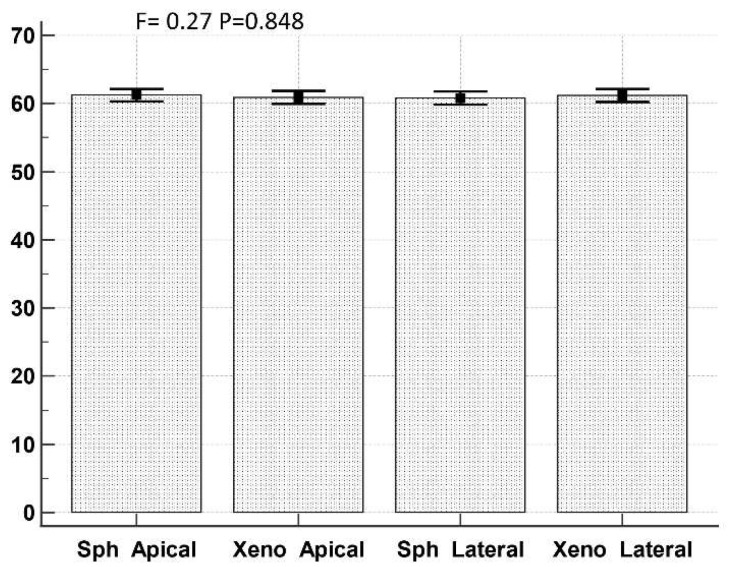
ANOVA test with Bonferroni correction of diameter values of apical-secreted and basolateral-secreted exosomes in MTSs and xenografts. Sph Apical: exosome diameter in spheroid apical secretion; Sph Lateral: exosome diameter in spheroid basolateral secretion; Xeno Apical: exosome diameter in xenograft apical secretion; Xeno Lateral: exosome diameter in xenograft basolateral secretion.

To verify if some difference exists in exosome diameter values in different samples (MTS vs. xenograft) and different secretion sites (apical vs. basolateral), an ANOVA test with Bonferroni correction was performed (Figure 12).

**Figure 13:**
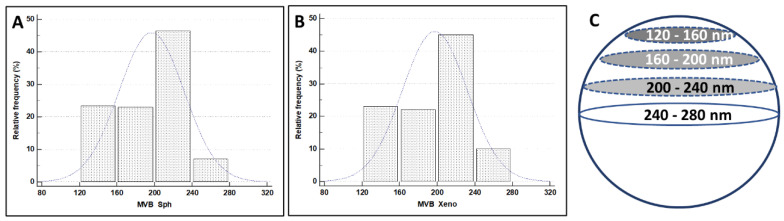
Histograms illustrate the distribution of MVB diameter values in MTSs and xenografts. (**A**) MVB diameter in spheroid secretion. (**B**) MVB diameter in xenograft secretion. (**C**) The maximum diameter value is the less frequent, and the others occur at least two times (over and above the equator).

The size of MVBs secreted from the apical domain of spheroid and xenograft was measured and compared. MVBs secreted by the spheroid basolateral domain were scarce (being present only in type C MTSs) concerning that of xenotransplant, and no comparison between these two groups was made. Results of statistical data analysis are reported above in Table 3 and Figure 13.

**Figure 14:**
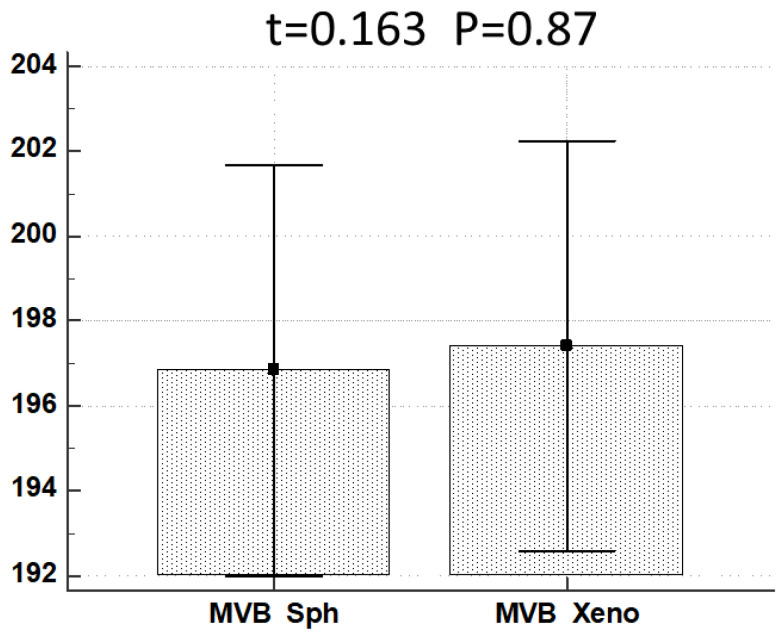
*t*-test results comparing MVB diameter in MTSs (MVB Sph) and xenografts (MVB Xeno).

To verify if some difference exists between MVB diameter value in MTSs vs. xenografts, a *t*-test was performed and results are presented in Figure 14.

## 4. Discussion

Cancer stem cells are a tumor subpopulation capable of self-renewal and are crucial for survival, proliferation, drug resistance, metastasis, and tumor recurrence [35]. We recently generated a molecularly characterized biobank of colorectal CSC-enriched lines that represent a priceless resource available for in vitro studies and for the development of CSC-based murine models that faithfully reproduce the molecular and histological features of the primary tumor [10]. This primary tridimensional cell culture of tumor-derived MTSs retains the genetic heterogeneity of the original patient tumor and displays CSC’s ability to dynamically switch between CSC and non-CSC states [35,36]. CRC spheroid cultures also reproduce drug sensitivity profiles of parental tumors, thus representing an excellent preclinical model to investigate the efficacy of new anticancer therapies [10,11]. While the use of MTSs for drug testing has been the object of intense studies, the biological and structural features of MTSs are less explored.

We have previously investigated the ultrastructural features of CRC MTSs, highlighting an increased presence of stem-like cells in MTSs as compared to tumor xenografts [24]. In this study, we investigated the presence and localization of exosomes in CRC MTSs and xenografts. Exosomes play a crucial role in mediating cell-to-cell communication between CSCs, non-stem cancer cells, and other cells in the tumor microenvironment (TME), regulating processes such as tumor progression, metastasis, drug resistance, EMT, and immune evasion [37]. The results of our ultrastructural study show that spheroids have exosome secretion patterns that depend on their structural complexity. Precisely, spheroids formed by a few layers of cells (Group A), with little adhesion between them, do not show exosomes production; when the number of cell layers increases and the degree of cell adhesion of the spheroid increases (Group B), secretion of exosomes into the lumen of pseudocysts is observed; until we have, in spheroids with several cell layers and a high degree of intercellular adhesion (Group C), secretion of exosomes and multivesicular bodies both from the apical domain of cells surrounding the lumen of the pseudocysts, both from the basolateral domain of the same cells and also from cells that do not face the lumen.

As reported in [38], MTSs from different CRC cell lines organize into three main types: loose, tight, and compact, and this different organization is related to different adhesion molecules expression. Loose MTSs express integrin-mediated interactions that are subsequently substituted by N-cadherin and, finally, E-cadherin interactions. In the study of [33,34,39], the different molecular profiles of apical vs. basolateral exosome secretion were demonstrated.

Our ultrastructural results (from a patient-derived CRC cell line) correlate for the first time the molecular data from the literature with the ultrastructural imaging of the different loose, tight, and compact MTS organizations, showing its relation with different exosome secretion patterns and amounts from both the apical and basolateral surfaces. This means that exosome production can be targeted in different pathways [40] to slow its progression. Lower exosome production will result in the lowering of cancer-promoting effects triggered by exosome-carried molecules.

In xenograft models (from a patient-derived MTSs xenograft), the secretion of exosomes occurs from all domains of the tumor cells and is quantitatively greater than that observed in spheroids. The massive presence of MVBs that release their contents into the lumen of the pseudocysts or in the tissue interstitium was observed.

Our ultrastructural results show for the first time the different arrangements in rows and columns (different patterns) and the difference in the amount of MBV secretion between spheroids and xenografts, xenografts being the source of a massive exosome and MVB production.

Our findings suggest that targeting the pathways of MVB formation and release could be another way to slow down cancer progression and translate our findings into clinical applications.

This difference in exosome secretion pattern and amount between MTSs and xenografts may be possibly due to the influence of surrounding non-tumor cells, as it has been shown that exosomes are key mediators of the communication between tumor cells and the tumor microenvironment [41].

Statistical analysis conducted on measurements of exosome diameter shows that exosomes secreted from the different MTSs domains have the same size (about 70 nm), which is then also the same in those produced by xenograft cells. According to [42], the size of our observed exosomes corresponds to that of a “small exosome” (Exo-S), ranging from 60 to 80 nm. Exo-S are particularly rich in Flotillin 1, flotillin 2, tweety family member 3, tetraspanin 14, and ESCRT-I subunit VPS37B. The data in [42] indicate that exosome size, in addition to their specific cargo, may influence metastatic patterning and the systemic effects of cancer.

## 5. Conclusions

Our morphological data show that structural complexity influences exosome secretion of MTSs, in both intensity and pattern, if compared with xenograft models. Our observations add new knowledge to the ultrastructural features of CRC MTSs and xenografts. Future studies may define the mechanistic basis of different exosome secretion patterns in the two model systems.

## Figures and Tables

**Table 1:** Summary statistic of MVBs number in apical and basolateral domains in MTSs and xenograft.

MBVs (200 Analyzed Cells for Each Group)	Arithmetic Mean	Std. Error	95% CI
MVBs Sph Apical	3.07	0.22	2.6272 to 3.5128
MVBs Sph Lateral	0.65	0.56	0.5504 to 0.7496
MVBs Xeno Apical	41.52	0.05	40.4102 to 42.6298
MVBs Xeno Lateral	3.94	0.09	3.7659 to 4.1241

MVBs Sph Apical: MVBs in the spheroid apical domain; MVBs Sph Lateral: MVBs in the spheroid basolateral domain; MVBs Xeno_Apical: MVBs in the xenograft apical domain; MVBs Xeno_Lateral: MVBs in the xenograft basolateral domain.

**Table 2:** Summary statistic of diameter values in apical-secreted and basolateral-secreted exosomes, in MTSs and xenograft.

Sample Diameter (N = 200 Each Group)	Lower Value (nm)	Higher Value (nm)	Mean	Std. Error	95% CI
Sph Apical	50	71	61.2637	0.4633	60.3502 to 62.1772
Sph Lateral	50	72	60.7761	0.4829	59.8240 to 61.7283
Xeno Apical	51	73	60.8756	0.4776	59. 9338 to 61.8174
Xeno Lateral	50	71	61.1841	0.4791	60.2394 to 62.1288

Sph Apical: exosome diameter in spheroid apical secretion; Sph Lateral: exosome diameter in spheroid basolateral secretion; Xeno Apical: exosome diameter in xenograft apical secretion; Xeno Lateral: exosome diameter in xenograft basolateral secretion.

**Table 3:** Summary statistic of MVB diameter values in MTSs and xenograft.

(N = 200 Each)	Lower Value (nm)	Higher Value (nm)	Mean	95% CI	Variance	Standard Deviation
MVB Sph	134	240	196.8550	192.0112–201.6988	1206.7377	34.7381
MVB Xeno	131	243	197.4200	192.5860–202.2540	1201.8529	34.6677

MVB_Sph: MVB diameter in spheroid; MVB Xeno: MVB diameter in xenograft.

## Data Availability

Datasets analyzed during the study are archived in the Human anatomy section of the Anatomical Histological, Forensic Medicine, and Orthopedic Sciences department of Sapienza University and are available upon request.
